# Pauper Lunatics and Imbeciles

**Published:** 1905-10-07

**Authors:** 


### PAUPER LUNATICS AND IMBECILES.

Under the above heading the *Times* of September 29 contained a sensible and interesting letter from Mr. W. H. Aubrey, who describes himself as a Guardian of the Croydon Union, and who enters a protest against the enormous expense incurred by the borough in the mere housing of its pauper lunatics, an expense which does not appear to be attended with any compensating or equivalent advantage, and which falls very heavily upon the general body of the ratepayers, many of whom are in anything but affluent circumstances, and are themselves housed in a manner very inferior to that of those whom they are compelled to support. Mr. Aubrey says that the Warlingham Hospital, to which the Croydon cases are sent, was completed two years ago, and that it then cost Croydon £700 merely to remove the patients. The site is three miles and a half from a railway station, a position which involved an enormous increase of cost in building, and which puts the friends of patients to much fatigue or expense in order to visit them. The land and buildings were to have cost £80,000, but the actual disbursements have amounted to nearly a quarter of a million, or about £555 for each inmate. The hospital has, he says, a mile or two of spacious corridors, constructed of glazed bricks and tiles. All the joinery work is of the best. The wards are some 18 feet in height, the floors are stained and varnished, the lofty windows, hundreds in number, are hung with curtains, the tables are covered with thick coloured cloths, and easy chairs and couches, of divers shapes and patterns, with any number of bent wood cane chairs, are provided. Each ward has a full-sized grand piano. As a general result, Mr. Aubrey says that each inmate of the Croydon workhouse costs 8s. a week, and each inmate of the hospital 22s. 2¾d. Each recipient of out-door relief costs 3s. 5d., each casual 1s. 5⅝d., and each child in a cottage home 10s. 6¼d. per week. Taking the country as a whole, it costs 17d. to disburse a shilling in actual relief; and, in spite of palatial asylums, insanity is steadily increasing. If less money were spent upon luxuries, to which the class receiving them are wholly unaccustomed, and more upon clinical and pathological laboratories, properly equipped and staffed, it is not improbable that the annual statistics would soon tell a tale different from the present one. It is greatly to be feared, moreover, that the note of extravagance in building and furnishing prevails in sanatoria as well as in asylums, and that the actual utility of both is likely to be thereby seriously impaired, and the matter is one which calls for the attention of contributors in the one case and of ratepayers in the other. In the *Times* of October 3 Mr. Charles Fox writes to comment on Mr. Aubrey's letter, and to point out that the Croydon ratepayers let the whole question of the Warlingham Hospital be decided without any appearance being made on their behalf. It is mainly to apathy of this kind that many municipal and other extravagances are to be attributed.

